# Weed Diversity Affects Soybean and Maize Yield in a Long Term Experiment in Michigan, USA

**DOI:** 10.3389/fpls.2017.00236

**Published:** 2017-02-24

**Authors:** Rosana Ferrero, Mauricio Lima, Adam S. Davis, Jose L. Gonzalez-Andujar

**Affiliations:** ^1^Departamento Protección de Cultivos, Instituto de Agricultura Sostenible, Consejo Superior de Investigaciones CientíficasCórdoba, Spain; ^2^Center of Applied Ecology and Sustainability (CAPES), Pontificia Universidad Católica de ChileSantiago, Chile; ^3^Facultad de Ciencias Biológicas, Pontificia Universidad Católica de ChileSantiago, Chile; ^4^Laboratorio Internacional de Cambio Global, (CSIC-PUC)Santiago, Chile; ^5^Global Change and Photosynthesis Research Unit, Agricultural Research Service (USDA)Urbana, IL, USA

**Keywords:** climate change, maize, long-term experiment, soybean, weed diversity, nonlinearity, crop management

## Abstract

Managing production environments in ways that promote weed community diversity may enhance both crop production and the development of a more sustainable agriculture. This study analyzed data of productivity of maize (corn) and soybean in plots in the Main Cropping System Experiment (MCSE) at the W. K. Kellogg Biological Station Long-Term Ecological Research (KBS-LTER) in Michigan, USA, from 1996 to 2011. We used models derived from population ecology to explore how weed diversity, temperature, and precipitation interact with crop yields. Using three types of models that considered internal and external (climate and weeds) factors, with additive or non-linear variants, we found that changes in weed diversity were associated with changes in rates of crop yield increase over time for both maize and soybeans. The intrinsic capacity for soybean yield increase in response to the environment was greater under more diverse weed communities. Soybean production risks were greatest in the least weed diverse systems, in which each weed species lost was associated with progressively greater crop yield losses. Managing for weed community diversity, while suppressing dominant, highly competitive weeds, may be a helpful strategy for supporting long term increases in soybean productivity. In maize, there was a negative and non-additive response of yields to the interaction between weed diversity and minimum air temperatures. When cold temperatures constrained potential maize productivity through limited resources, negative interactions with weed diversity became more pronounced. We suggest that: (1) maize was less competitive in cold years allowing higher weed diversity and the dominance of some weed species; or (2) that cold years resulted in increased weed richness and prevalence of competitive weeds, thus reducing crop yields. Therefore, we propose to control dominant weed species especially in the years of low yield and extreme minimum temperatures to improve maize yields. Results of our study indicate that through the proactive management of weed diversity, it may be possible to promote both high productivity of crops and environmental sustainability.

## Introduction

Feeding more people sustainably is among humanity's biggest challenges in the next century (Godfray et al., 2010). Until now, agricultural expansion and intensification has had tremendous impacts including environmental degradation from loss of biodiversity and habitat, chemical inputs into waterways and deterioration of soil health (Foley et al., 2011). As agricultural production must increase by at least 60% before 2050 to meet increasing population and consumption trends (Alexandratos and Bruinsma, 2012), doing so in ways that do not compromise environmental integrity or public health will be a great challenge (Tilman et al., 2002). One promising approach to sustainable intensification is to assess, and make use of agroecosystem services that enhanced biodiversity might provide (Vandermeer et al., 1998; Swift et al., 2004; Cardinale et al., 2012; Robertson et al., 2014). However, we must first seek to understand how agricultural systems are related to biodiversity, to develop new strategies that take advantage of ecological interactions within agricultural systems (Loreau et al., 2001). Here we explore the potential effects of diversifying weed community systems as a means of controlling harmful species while simultaneously enhancing desirable agroecosystem services. Opportunities to develop strategies of weed community management based on differences in weed diversity exist to enhance crop production and producing a more sustainable agriculture.

Weeds compete with crop species, causing total crop losses in some cases, but also play an important role in supporting agroecosystem functions and services. For example, weeds can enhance soil and water conservation, nutrient cycling, pollination activity, provide food sources for animals and insects, and host biological control agents (Altieri, 1999; Marshall and Brown, 2003). Recent findings suggest the importance of weed diversity within agroecosystems, and highlight new options for weed management (Franke and Lotz, 2009; Petit and Boursault, 2010) that balance yield and weed diversity conservation. However, very little is known about how weed community diversity is related to crop yield and its temporal variability. Mechanistic studies are needed to make reliable recommendations (Davis et al., 2005), and such studies should take into account several important considerations. First, climate change and climate variability may underlie and confound the diversity–productivity relationship (Vilà et al., 2005; Belote et al., 2011). Second, the effects of biodiversity on ecosystem functioning appear to vary over time and space (Mittelbach et al., 2001; Symstad et al., 2003). Finally, management inferences drawn from such investigations should strike a balance between adequate weed control and the requirements for biodiversity and more sustainable production methods (Marshall and Brown, 2003).

Here we study maize and soybean yield in the Main Cropping System Experiment (*MCSE*) from 1996 to 2011 at the Long-Term Ecological Research site at the W. K. Kellogg Biological Station (*KBS LTER*) located in southwest Michigan. KBS is the only LTER site focused on row-crop agriculture and represents one of the most extensive and important agroecosystem types (Smith et al., 2008) in the USA that is managed intensively with well-known environmental impacts (Robertson and Hamilton, 2015). Extreme heat limits maize and soybean production in the region (Schlenker and Roberts, 2009; Mourtzinis et al., 2015; Leng et al., 2016) through increased water stress (Lobell and Asner, 2003; Lobell et al., 2013) and crop density (Lobell et al., 2014; Ort and Long, 2014). However, increases in minimum air temperature could be more significant in their effect on maize (Muchow et al., 1990; Chen et al., 2011; Grassini et al., 2011; Hatfield et al., 2011). Among US expenditures to ameliorate biotic stresses in agriculture, weeds are the most costly.

It has been suggested that we need to know how to manage cropland for an array of ecosystem services to balance or reduce the negative impacts of agricultural production, an area of research largely unexplored (Robertson and Hamilton, 2015). In this study, we evaluate the role of weed diversity in supporting agricultural productivity and sustainability in cropping systems. For this purpose we will use an analytical tool we have recently proposed based on population dynamic theory to investigate crop yield oscillations (Ferrero et al., 2014). While not a true population in the reproductive sense, cropping systems obey similar rules as other dynamic systems, both natural and engineered (Ferrero et al., 2014). This method allows us to include logical explanations of the possible effects of weed community diversity on the rates of yield increase under climate perturbations.

## Materials and methods

### Empirical databases and data preprocessing

**Figure 1:**
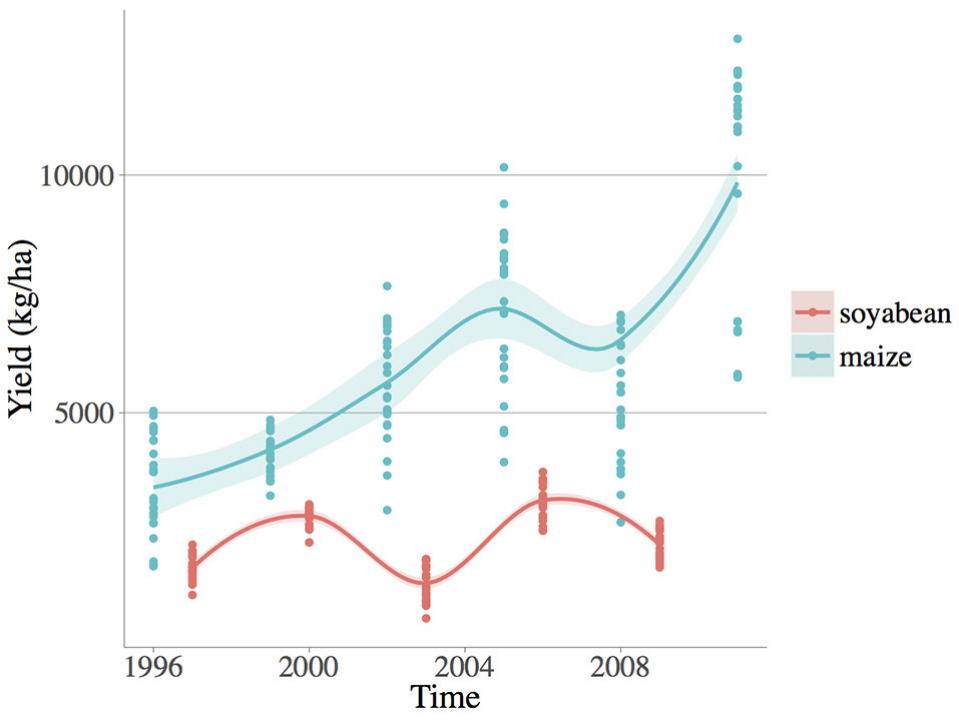
**Observed yield numerical fluctuations (***kg/ha***) of the two crop species: soybean (***Glycine max*** [L.] Merr.) and maize (***Zea mays*** L.)**. Loess smoothed fit curves with confidence regions are showed.

A time series of crop, weed, and site climate data were obtained with permission from the Main Cropping System Experiment (*MCSE*) of the Kellogg Biological Station's Long-Term Ecological Research (*KBS-LTER*) site in southwest Michigan, USA (Smith et al., 2008; http://lter.kbs.msu.edu/datatables/). From 1996 to 2011, the annual cropping systems were maize (*Zea mays* L.) -soybean (*Glycine max* [L.] Merr.) -winter wheat (*Triticum aestivum* L.) rotations under four different management regimes (Figure 1). One system (T1) was managed conventionally on the basis of current cropping practices in the region with high external chemical input and tillage. A second (T2) was managed as no-till system, otherwise identical to the conventional system (high external chemical input but no tillage). A third (T3) was managed as a reduced-input system, receiving one-third of the conventional system's chemical inputs. A fourth system (T4) was managed organically with no external chemical inputs. The T3 and T4 systems were tilled in the same way as T1, but used winter cover crops of red clover or annual rye to provide additional nitrogen. Weed control was accomplished with herbicides (glyphosate) in T1, T2, and T3 (before 2009) and through additional physical cultivation in the T3 and T4 systems. Starting in 2009 (for soybean) and 2011 (for maize), transgenic crop cultivars were used, with glyphosate resistance and (for maize) resistance to European corn borer (*Ostrinia nubilalis*) and root worm (*Diabrotica* spp.). No transgenic crop cultivars were used in T4.

Each management system was replicated in 6 blocks of 1 ha in a randomized complete block design. Crop yields (i.e., crop kernel/seed harvested at crop harvest in *kg/ha*) were determined annually by harvesting each block at standardized moisture for maize 15.5% and wheat/soybean 13%. In this paper grain yield of maize and soybean were selected because they share a similar growing season, critical to our comparison of weed diversity and climate variability effects on crop production for similar agricultural situations. Maize (grown in 1996, 1999, 2002, 2005, 2008, and 2011) and soybean (grown in 1997, 2000^1^, 2003, 2006, and 2009) were harvested in November and October, respectively (see Table S1).

Weed biomass at the species level was measured at peak biomass for a given treatment with six replications (blocks). Plants were hand harvested using a 1 m^2^ quadrat at each sampling station before harvest of the crop plants. Weed biomass was separated to the species level, dried to constant weight at 65°C, and dry biomass for each species recorded. Productivity data for plants other than target crops (i.e., weeds) were obtained as dry biomass m^−2^. We argue that crop biomass is more adequate than counts of individuals of variable sizes for measure plant abundance (Oksanen, 2013). For each crop, we derived the growing season average weed community richness and diversity across all samples using Richness, Shannon–Wiener, Simpson's, Inverse Simpson, *J*- and *E*-evenness indices. Details of how biodiversity indices were obtained are in the supplementary information, Supplementary material (Appendix A1). We used the *Biodiversity R* package (Kindt and Coe, 2005) implemented in the R CRAN environment (R Development Core Team, 2011) and applied the “diversityresult” function.

Daily precipitation (*mm*) and air temperature (°*C*) values were obtained from the KBS LTER Site Weather Station located in the experimental site (http://lter.kbs.msu.edu/datatables/). For each crop and sample, we derived the growing season average temperature (*tM*), the average maximum (*tmax*), and minimum (*tmin*) temperature, the average rainfall (*precM*) and extremes values (*tMax, tMin*, and *precMax*). Maize was planted early May and harvested late September; and soybean, planted late May and harvested early October.

### Diagnosis and models of crop yield dynamics

Crop yield dynamics are the result of the combined effect of internal (ecophysiological and biophysical traits related to resource acquisition and use, and the trade-offs between them that constrain crop production) and external (e.g., weed diversity and climate perturbations) processes (Ferrero et al., 2014). To understand how these processes may determine crop yield fluctuations, we analyze both as a general model based on the rates of yield increase.

#### Detection of internal processes

To minimize the influence of slowly changing factors (non-climatic influences) such as crop management and improvement in crop genetics, we detrended crop yield data by a quadratic trend (see Lobell et al., 2011b). Then, to generate a stationary time series we computed the first difference log-yield series (as in Lobell et al., 2011a,b). We used the difference in values for a given crop from one time step to the next value (in log-scale) and called it the crop yield rate of increase, *R*_*t*_ = *Y*_*t*_ − _*Y*_*t*_−1_.

A crop yield model incorporating both internal and external processes may be depicted as $R_{t}=f\left(Y_{t-d},Z_{t-d^{\prime }},\varepsilon_{t}\right)$, where *d* denotes the number of lags to be included and $Z_{t-d^{\prime }}$ is the weed diversity and/or climatic conditions (with lags *d* = 0 and 1). We used the Royama-type non-linear log-model (Ricker, 1954; Royama, 1992) as our form for the function *f* to fit time series data:

(1) $$\begin{array}{r} R_{t}=r_{\text{max}}-\text{exp}\left(aY_{t-d}+c\right) \end{array}$$

where *Y*_*t*−*d*_ represent crop yield at time *t* − *d*, *r*_max_ is a positive constant representing the maximum increase in crop yields (and is estimated as the maximum value observed from the data), *c* is a measure of the ratio between supply and demand of limiting resources and *a* is a shape parameter representing the non-linear interaction strengths (i.e., functional responses). To estimate the order of the process *d* we used the partial rate correlation function (*PRCF*; the partial correlation coefficients between *R*_*t*_ and *Y*_*t*−*i*_; Berryman and Turchin, 2001). This function is flexible enough to detect a wide range of system behaviors and includes a biologically realistic property: the crop performance is bounded (Royama, 1992), because no crop can produce an infinite yield value, there must be an upper bound in *R*_*t*_ in (1). Because in this model the three parameters *r*_max_, *c*, and *a* have an explicit biological interpretation we can include external perturbations in each parameter using the framework of Royama (1992). Thus, we can build biological hypotheses about the effects of climate and weed diversity on crop yields, and evaluates the consequences on the nontrivial root *R*_*t*_ = *K* (its equilibrium or yield potential) and the slope^2^ at the yield potential, which reflects the stability of the dynamic.

#### External perturbations: weed diversity and climate

External perturbations could translate the conditional function curve (Equation 1) with dynamic consequences (Appendix A2). We used the function *b*log$\left(Z_{t-d^{\prime }}\right)$ to represent perturbations of an external factor *Z* over a limited range of variation (Lobell and Burke, 2010) in each parameter of (1). In this manner, changes in *c* have non-additive effects on crop growth rates (lateral model, sensu Royama, 1992):

(2) $$\begin{array}{r} R_{t}=r_{\max}-\exp\left(aY_{t-d}+c+b\log\left(Z_{t-d^{\prime }}\right)\right) \end{array}$$

where $Z_{t-d^{\prime }}$ is the weed diversity and/or climatic conditions (with lags *d* = 0 and 1). For example, precipitation may influence the temporal variation of water availability, a limiting resource for crops. Here an external factor affects the yield potential but not its stability through changes in the amplitude or period of oscillation. The stability of the yield potential point is determined by the slope of the function in its vicinity (estimated by the product of *r*_max_ and *a*): the steeper the slope, the less stable the equilibrium (Berryman, 1999).

Simple additive effects occur when an external perturbation changes the maximum capacity of response of the crop (changes in *r*_max_; vertical model, sensu Royama, 1992):

(3) $$\begin{array}{r} R_{t}=r_{\max}-\exp\left(aY_{t-d}+c\right)+b\log\left(Z_{t-d^{\prime }}\right) \end{array}$$

That is, for example, when during warmer years the crop acquires less resources and produces less seeds than during colder years. Variation in this parameter could modify the equilibrium point and could alter its stability.

Changes in the nonlinearity parameter *a* represent a different functional response of growth rates to an external factor (non-linear model, sensu Royama, 1992):

(4) $$\begin{array}{r} R_{t}=r_{\max}-\exp\left(\left(a+b\log\left(Z_{t-d^{\prime }}\right)\right)Y_{t-d}+c\right) \end{array}$$

Here, for example, maximum temperature would affect water availability for crops according to some function for low temperature values, and according to another function for high values of temperature. This also could affect both the equilibrium point and its stability.

Finally, we deal with combined effects and non-independence of predictors by including interaction terms in each of these models (*b*log$\left(Z_{t-d^{\prime }}^{1}/Z_{t-d^{\prime }}^{2}\right)$).

In Appendix A2 we demonstrate how changes in *r*_max_ and *c* translate the conditional function curve *R*_*t*_ = *f*(*Y*_*t*−*d*_, *Z*) moving it vertically and laterally, respectively, but do not influence its relative shape (Royama, 1992). However, perturbations in *a* influence the relative shape of the conditional production curve.

#### Model fitting

We fitted Equations 2–4 using nonlinear least squares regressions with the *nls* library of the *R* statistical computing environment (Bates and Chambers, 1991; R Development Core Team, 2011). A model selection criterion was used to rank competing models and to weigh the relative support for each one. We used the multi-model selection methods described by Burnham et al. (2002) to compare a suite of candidate models using Akaike weights, *w*_*i*_. Akaike weights can be interpreted as the probability that model *i* is the best one for the observed data, given the candidate set of models (Johnson and Omland, 2004). We report confidence sets of models fitted to each data set, that is, the smallest subset of candidate models for which the *w*_*i*_ sum to 0.99. We reported also the pseudo *R*^2^ measures based on the deviance residual (Cameron and Windmeijer, 1996).

#### Analysis of temporal diversity

When we detected an association between crop yield and weed community diversity, we identified the weed species that contribute most to these diversity indexes to focus on more important components of the weed community. Weed database were reduced to eliminate rare species (<5% of a sample in entire data set) and avoid unduly large influence on the analysis. Pearson's correlation coefficients were computed between the weed diversity indices and the Hellinger-transformed weed species data (a standardization method; Legendre and Gallagher, 2001). The large positive/negative correlations identified the weed species that contributed more to the observations with large/small weed diversity indices. We used a threshold of |*r*|>0.4 for inclusion in correlations of interest. These correlations could not be tested for significance because the diversity indices were not independent from the species data from which were computed.

## Results

**Figure 2:**
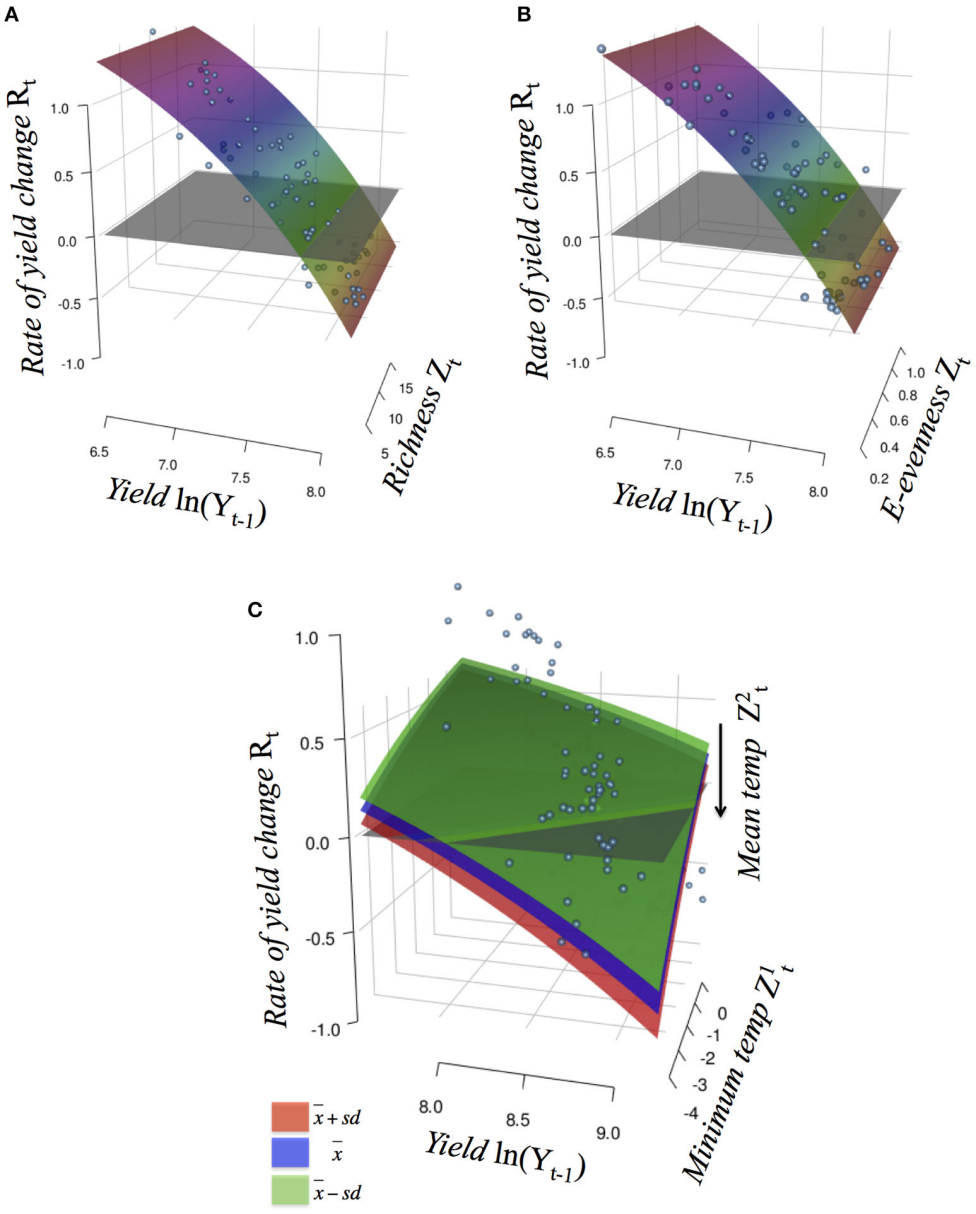
**Crop yield rates of change ***R**_**t**_* against the log observed yield level ***Y***_***t*****−1**_ (with 1 year of delay) for soybean (***GlycL***), with additive effects of the external factor ***Z***_***t***_ that perturbs the productivity function (***R***-function): (A)** richness and **(B)** E-evenness; and for maize (*ZeaL*) with non-additive perturbations of the interaction between **(C)** minimum and average temperature (*tMin* and *tM*, respectively). In **(A, B)** colors indicate the Rt values; in **(C)** colors indicate categories for mean temperature values. See Table S1 for description of models and Figure S2 for their graphs.

Detrended time series of soybean and maize yield exhibited first-order negative feedback structures [*PRCF(1)*] as the most important component of yield growth rate (Figure S1). Due to these stabilizing feedbacks, both crops exhibited stable approaches to equilibrium (regular oscillations). Internal processes appeared to contribute more to the dynamics of soybean (*R*^2^ = 0.76, *AICc* = 61.67) than of maize (*R*^2^ = 0.49, *AICc* = −88.26; Table 1). Model selection retained 6 plausible models for soybean and 4 plausible models for maize (i.e., a 99% confidence set of models; Table 1). For soybean, all retained models included a positive weed community diversity effect (richness, evenness and diversity measures) on *r*_max_, which improved the explained variance by 20% (*R*^2^ = 0.96, *AICc* = −64.27 for the best model; Table 1, Figure 2, and Figure S2). Therefore, weed diversity affects the rate of crop yield change independently of the current yield level (additive). Although the rate of yield change increased, the dynamics of soybean production maintained a stable and damped approach to equilibrium when weed diversity was taken into account (Table 2).

**Table 1:** **Optimal crop yield models for maize (***ZeaL***) and soybean (***GlycL***) production using the exponential form of logistic growth**.

**Crop**	**Fit**	**Variable**	***r*****_max_**	***a***	***c***	***b***	***AIC*****_*c*_**	***ΔAIC*****_*c*_**	***w*****_*i*_**	***R*****^2^**
ZeaL	L	tM/tMin	1.20	0.37^**^	−5.49^**^	12.58^**^	−22.03	0	0.97	0.80
	L	tmax/tMin	1.20	0.41^**^	−5.99^**^	10.32^**^	−12.12	9.91	0.01	0.78
	L	Shannon/tMin	1.20	0.53^**^	−4.76^**^	11.87^**^	−11.83	10.19	0.01	0.84
	L	Simpson/tMin	1.20	0.51	−4.55^**^	12.85^**^	−11.76	10.26	0.01	0.84
	P		1.20	0.93^**^	−7.91^**^		88.26	87.27	0	0.49
GlycL	V	Eevennes	0.97	0.63^**^	−4.09^*^	0.31^*^	−64.27	0	0.22	0.96
	V	Simpson	0.97	0.64^**^	−4.15^*^	0.31^*^	−64.11	0.16	0.20	0.96
	V	Shannon	0.97	0.65^**^	−4.19^*^	0.30^*^	−63.98	0.30	0.19	0.96
	V	Invsimp	0.97	0.65^**^	−4.27^*^	0.30^*^	−63.73	0.54	0.17	0.96
	V	Jevenness	0.97	0.64^**^	−4.11^*^	0.31^*^	−63.15	1.12	0.13	0.97
	V	Richness	0.97	0.71^**^	−4.80^**^	0.26^*^	−62.34	1.93	0.08	0.96
	P		0.97	1.95^**^	−15.23^**^		61.67	125.95	0	0.76

*The best models were chosen by using multi-model selection methods described by Burnham et al. (2002) that compare a suite of candidate models using Akaike weights. We evaluated our basic model with internal processes (P) and the external perturbations of their parameters: r_max_ maximum finite reproductive rate (vertical model, V), a non-linearity coefficient (non-linear model, N), c the ratio between demand and offer of limiting resources (lateral model, L); with b coefficients for different external effects, AIC_c_ corrected Akaike information criterion, ΔAIC_c_ delta AIC_c_ and w_i_ Akaike weights of a set of selected models, and R^2^ pseudo-coefficient of determination. Further description of the variables included in the models are given under “Materials and Methods” (here minimum -tMin-, mean -tM- and average maximum -tmax- temperature for growing season and weed community diversity indices –Shannon and Simpson indices-)*.

** p < 0.01;

* *p < 0.05*.

**Table 2:** Descriptive statistics for climate and weed diversity variables for maize (***ZeaL***) and soybean (***GlycL***) production, where M, mean; SD, standard deviation; R, range; N, size and the percent of change in model parameters: for slope (it is estimated by ***r***_max_^*^a), yield potential ***K***, and the maximum increase in crop yields ***r***_**max**_.

**Crop**	**Fit**	**Variable**	**M**	**SD**	**R**	**N**	**slope**	**% slope**	**% ***r***_max_**	**% ***K*****
ZeaL	L	tM/tMin	−14.18	252.60	(−479.19; 388.53)	118	0.44	0.00	0.00	−16.30
	L	tmax/tMin	−17.31	335.89	(−633.27; 521.03)	118	0.49			−8.28
	L	Shannon/tMin	1.21	18.06	(−45.07; 41.03)	92	0.64			−8.07
	L	Simpson/tMin	0.46	8.53	(−20.02; 15.85)	92	0.61			−8.18
	P					118	1.12			
GlycL	V	Eevenness	0.52	0.25	(0.16; 1)	71	1.51	0.11	0.11	0.02
	V	Simpson	0.40	0.23	(0; 0.83)	71	1.54	0.11	0.11	0.02
	V	Shannon	0.78	0.46	(0; 2.07)	71	1.53	0.26	0.26	0.05
	V	Invsimp	1.99	0.89	(1; 5.90)	71	1.53	0.60	0.60	0.12
	V	Jevenness	0.51	0.21	(0.04; 0.99)	69	1.54	0.12	1.22	0.02
	V	Richness	5.93	3.80	(1; 18)	71	1.56	1.86	1.86	0.33
	P					94	1.89			

*See Table 1 for more details*.

**Figure 3:**
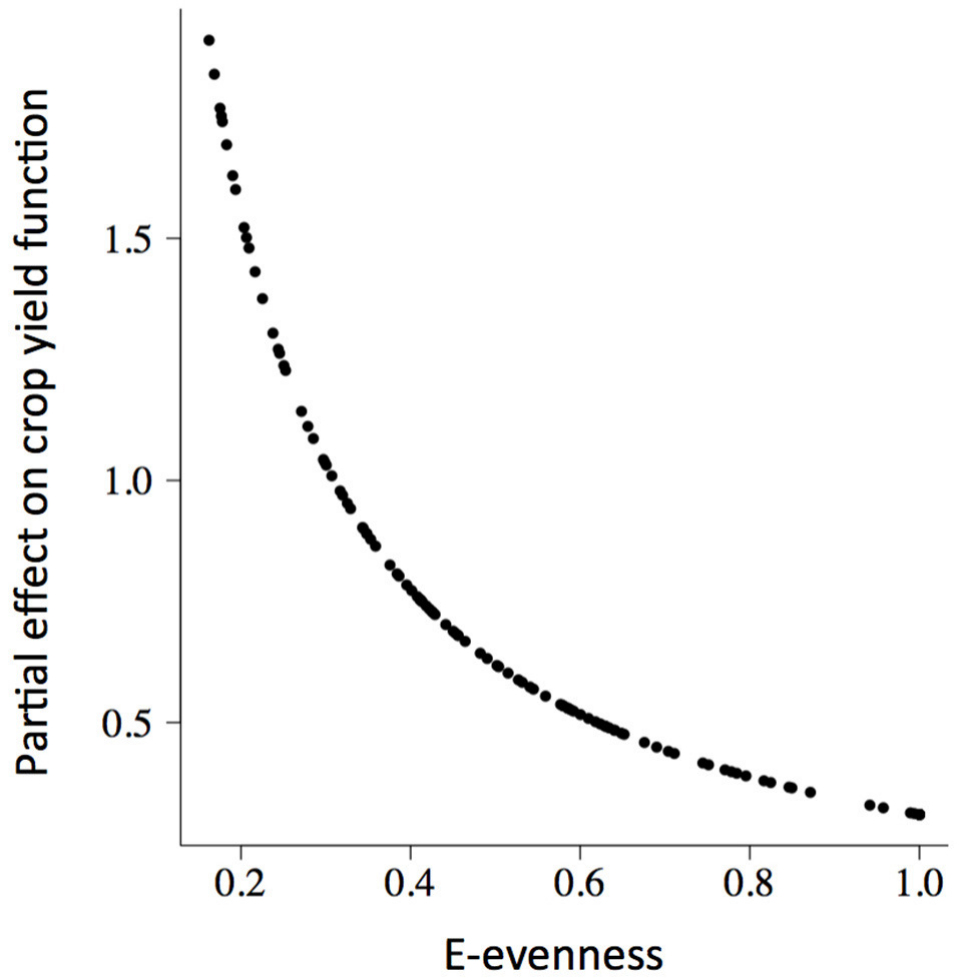
**The partial slope of the fitted soybean (***GlycL***) production surface (δ***R***_***t***_/δ***Z***_***t***_) with respect to the weed E-evenness of plots (***Z***_***t***_)**. There was a positive relationship between weed community diversity and soybean production, with a deceleration as the agro-ecosystem moved to a more diverse system.

The rate of change of soybean growth yields was decelerated as the agro-ecosystem moved to a more diverse system (holding yield fixed; partial derivative ∂*R*_*t*_/∂*Z* = *b*/*Z*; for example Figure 3 for Evenness). This is consistent with the low values of percentage of increase found for the model parameters (Table 2). For soybean, high weed Evenness values were often related to a small number of weed species (*r* = −0.73; Figure S3). Red clover (*Trifolium pratense* L., “Michigan Mammoth Red,” TRIPR; Figure S4) biomass was positively correlated with the inverse Simpson index in reduced input and organic plots. Weed richness was positively correlated with garden yellowrocket (*Barbarea vulgaris* R. Br., BARVU) and shepherd's purse biomass (*Capsella bursa-pastoris* L., CAPBU), both winter annuals and rare species; negatively correlated with large crabgrass (*Digitaria sanguinalis* L., DIGSA; a summer annual species) biomass and positively but less to giant foxtail biomass (*Setaria faberi* L., SETFA; a highly adaptable, quick-recruiting summer annual species).

For maize, the 4 best-performing models included negative effects on *c* of extreme minimum temperature (*tMin*) through interaction with: average temperature (*tM*; *w*_*i*_ = 0.97), average maximum temperature (*tmax*; *w*_*i*_ = 0.01), Shannon diversity index (*shannon*; *w*_*i*_ = 0.01) and Simpson diversity index (*simpson*; *w*_*i*_ = 0.01); with more than 78% of variance explained (and $R^{2}=0.80,AIC_{c}=-22.03$ for the best model; Table 2). As an interaction effect is present, the impact of extreme minimum temperature depends on the level of the other variable. All models suggested that less negative minimum temperatures act to increase maize production, whereas mean and maximum temperature, as well as weed diversity, constrains this yield potential (Figure 2 and Figure S2).

For maize, weed Shannon, and Simpson diversity was negatively correlated to weed evenness (*r* = −0.36 and −0.30, respectively; Figure S3). As show in Figure S5, weed diversity was positively correlated with common dandelion (*Taraxacum officinale*, TAROF; winter perennial species), lambsquarters (*Chenopodium album* L., CHEAL; summer annual species) and velvetleaf (*Abutilon theophrasti* Medik, ABUTH; summer annual species) biomass. All are important weeds of field maize production systems in the U.S. Corn Belt.

## Discussion

In our long-term analysis (1996–2011) we showed that the combined effects of internal and external processes involving weed diversity were strongly associated with soybean and maize yield fluctuations. Internal processes included ecophysiological and biophysical traits related to resource acquisition and use, and the trade-offs between them (by first-order negative feedbacks) that constrain crop production and produced rapid and stable yield fluctuations, consistent with our previous study of maize in Spain (Ferrero et al., 2014). The regulation of crop production appears to be stronger in soybean (76%) than maize (49%; “P” line in Table 1) and therefore maize seems to be more sensitive to environmental variation. Increases in weed diversity were significantly associated with soybean and maize yield, but these relationships were crop-species specific. In soybean, yield increased additively with weed diversity, whereas increasing weed diversity constrained maize yield non-additively under freezing temperatures. For soybean this result implies that under higher weed diversity, the crops additively improved its maximum capacity of response (changes in *r*_max_). Namely, during years of higher weed diversity, soybean acquires more resources and/or produces more seeds than during years of lower weed diversity, thus affecting the intrinsic capacity of increase in soybean yields (*r*_max_; a species-specific adaptation). Under conditions favoring high weed diversity, soybean may be more adapted to resist yield decline due to negative environmental effects. For example, weeds could alter the crop environment changing the light interception, critical weed-free period for soybean or through allelopathic effects (Morvillo et al., 2011). Identifying which functional traits underlie weed diversity effects, and also characterizing these traits in relation to the ecology of each crop, may be an important area for future research.

This positive association between soybean yield and weed diversity appears particularly relevant to low-diversity agoecosystems, typical of grain production systems in the northern Corn Belt of the USA. The increases in crop production accelerated at low weed diversity values (Table 2, Figure 3), and then, in low-diversity plots small increases in weed diversity are sufficient to enhance crop productivity. Because the moderate correlation between weed evenness and the others diversity indexes, we could conclude that the effect of weed community is not only due to species identity but also to species interactions (i.e., complementarity and facilitation) maybe as supporters of reliable ecosystem functioning (Naeem, 2008). Put another way, the hyperbolic relationship we observed indicated that each additional species lost from our agroecosystem had a progressively greater negative impact on crop production. Therefore, the greatest crop production risks were in the least diverse systems. Also, weed diversity effects on soybean production did not change the stability properties of crop yield dynamics (amplitude and periodicity) and therefore did not provide support for the predictions of lower crop yield stability proposed by models of resource competition (Tilman, 1999). The potential benefits that weeds provide to cropping systems may help identify opportunities to harness their beneficial effects and to develop better weed management strategies (Smith et al., 2011). We hypothesized that the improved capacity to achieve high levels of productivity would promote an economically profitable system with a reduced need for external inputs as was proposed for increasing cropping system diversity (Davis et al., 2012). We call for future experiments explicitly to evaluate these potential links.

For maize, interaction terms of extreme minimum temperature with mean and maximum temperature, as well as weed diversity indices, negative, and non-additively affected maize yield through changes in the supply and/or demand of some limiting resources (changes in *c*; Table 1, Figure 2, and Figure S2). This implies that the effects of these variables must be considered in conjunction with constraints on the yield potential of maize, and indicate that the negative impacts of extreme minimum temperature increase with increasing levels of *tM* or *tmax*, and weed *Shannon* and *Simpson* diversity. The first finding is in agreement with a previous study on US maize where there was a large negative response of maize yields to increased temperature range, as a result of greater water and heat stress during hot days and crop injury or death on freezing night temperatures (Lobell, 2007). This result also supports previous studies showing that frost periods at either end of the growing season were crucial for maize production (Miedema, 1982; Muchow, 1990; Lobell, 2007; Chen et al., 2011; Ferrero et al., 2014) at this high latitude site. The second finding indicates that negative effects of weed diversity on maize yield are mediated through decreases in the minimum temperature.

Including the interactions between exogenous factors allowed us to model concurrent and compound effects on crop yields. As a result, we were able to detect that the impact of weed diversity on crop yields was more pronounced when coupled with lower temperatures (cooler nights). This result has major implications for the accuracy and reliability of crop yield forecasting models and decision-support, because it might not have been revealed (or confused) if we had only analyzed the exogenous factors separately. In explaining the above results, we suggest that: (1) maize was less competitive in cold years allowing higher weed diversity and the dominance of some weed species; or (2) that cold years resulted in increased weed richness and prevalence of competitive weeds, thus reducing crop yields. Further studies are necessary to clarify these mechanisms, although our findings indicate the need to control undesirable, highly competitive species like common dandelion, lambsquarters, and velvetleaf within a maize crop. Finally, our results differ somewhat from other studies in which plant species diversity impacts on crop yields were analyzed with respect to crop sequence diversification; for example, Smith et al. (2008) indicated that maize grain yield increased linearly in response to the diversity of the system, specifically the number of crops in the rotation. However, here we have found that small changes in weed diversity and minimum temperature interaction could exert big changes in maize production when yield levels are low, due to nonlinear responses. Such information can aid crop yield forecasting, preventing overly optimistic predictions by recognizing that these forecasting tools may not be equally effective at low and high crop yield levels.

Our findings complement the results from previous studies that assessed the impact of diversity on grassland productivity (Hector et al., 1999; Tilman et al., 2001, 1996) for two reasons. First, by improving the debate over biodiversity and ecosystem functioning, showing the role of weed diversity on crop productivity. We suggest that weed diversity could also play an important role in supporting agroecosystem functions and services in crop systems. Second, differentiating additive and non-additive effects that act through different potential mechanisms. We found a higher soybean rate of yield increase (and its potential yield) for weed communities more evenness, and that under lower temperatures (cooler nights) weeds could harm maize when it is at high yield levels.

## Conclusions

The results presented here show that the conditions underlying weed diversity in soybean and maize fields can lead to significant increases in crop yields. The new analytical approach presented here elucidates how crops are related to weed biodiversity loss or gain, and detects the minimum level of biodiversity associated with stable, bountiful crop yields. A better understanding of little-observed structural properties of agroecosystems, such as the stability of yield dynamics and nonlinear responses to weed diversity and climate variability observed in this analysis, can help guide management practices to maintain crop productivity under increased environmental variability through judicious management of biodiversity in agroecosystems. While testing the specific mechanisms is beyond the scope of our analysis, our results should motivate future studies to evaluate these potential links.

## Data accessibility

Crop yield data are available at http://lter.kbs.msu.edu/datatables/51 and correspond to the rotation of 3 crops (maize-soybean-winter wheat), with 7 treatments (T1 …T7) of which we only use the first 4: traditional management (T1), conservation management (T2 and T3) and ecological management (T4). Each treatment has 6 replicates. Weed biomass data (http://lter.kbs.msu.edu/datatables/40), daily precipitation and air temperature (http://lter.kbs.msu.edu/datatables/7) were also obtained for each treatment and replicate.

## Author contributions

All authors conceived the ideas, designed methodology and contributed critically to the drafts and gave final approval for publication; AD collected the data; RF analyzed the data and led the writing of the manuscript.

### Conflict of interest statement

The authors declare that the research was conducted in the absence of any commercial or financial relationships that could be construed as a potential conflict of interest.

## Footnotes

^1^There were missing data for weeds in the year 2000.

^2^The stability of the equilibrium point is determined by the slope of the *R*-function in the vicinity of equilibrium ($r_{\text{max}}^{*}a$): the steeper the slope, the less stable the equilibrium (Royama, 1992).

## Abbreviations

Large crabgrass: *Digitaria sanguinalis* L. DIGSA
red clover: *Trifolium pratense* L. “Michigan Mammoth Red TRIPR
garden yellowrocket: *Barbarea vulgaris* R. Br. BARVU
shepherd's purse: *Capsella bursa-pastoris* L. CAPBU
giant foxtail: *Setaria faberi* L. SETFA
lambsquarters: *Chenopodium album* L. CHEAL
common dandelion: *Taraxacum officinale* TAROF
velvetleaf: *Abutilon theophrasti* Medik ABUTH
soybean: *Glycine max* [L.] Merr.
and maize: *Zea mays* L.
