# Supplementary material for: Weed Diversity Affects Soybean and Maize Yield in a Long Term Experiment in Michigan, USA
Source: Front Plant Sci. 2017 Feb 24;8:236. doi: 10.3389/fpls.2017.00236 (PMC5323402; doi:10.3389/fpls.2017.00236)
Supplement: Supplementary file 1 [file Table1.DOCX]

**Supplementary information**

**Table S1.** Descriptive statistics for crop yield (kg/ha), precipitation (mm), temperature (degrees C) and weed diversity (based on weed biomass, g/m^2^) variables for maize (*ZeaL*) and soyabean (*GlycL*) production, 1996–2011. Mean (*pM*) and maximum (*pMax*) precipitation, minimum (*tMin*), maximum (*tMin*) and mean (*tM*) temperature, the average minimum (*tmin*) and maximum (*tmax*) temperature for growing season and weed community diversity indices –*Shannon,* *Simpson*, *Inverse Simpson, J* and *E evenness* indices-. See SI Appendix A1 for biodiversity indices explanation.

|  | *GlycL*, N=141 | | | | | *ZeaL*, N=144 | | | | |
| --- | --- | --- | --- | --- | --- | --- | --- | --- | --- | --- |
| Variable | Min | Max | M | SD | Md | Min | Max | M | SD | Md |
| *Yield* | 681.90 | 3758.30 | 2277.39 | 718.19 | 2250.15 | 1778.00 | 12861.50 | 5990.04 | 2648.84 | 5102.65 |
| *Shannon* | 0.00 | 2.07 | 0.78 | 0.46 | 0.76 | 0.00 | 1.93 | 0.90 | 0.56 | 0.92 |
| *Simpson* | 0.00 | 0.83 | 0.40 | 0.23 | 0.42 | 0.00 | 0.84 | 0.44 | 0.26 | 0.48 |
| *Invsimpson* | 1.00 | 5.90 | 1.99 | 0.89 | 1.72 | 1.00 | 6.11 | 2.25 | 1.14 | 1.93 |
| *J-evenness* | 0.04 | 0.99 | 0.51 | 0.21 | 0.52 | 0.03 | 1.00 | 0.49 | 0.21 | 0.51 |
| *E-evenness* | 0.16 | 1.00 | 0.52 | 0.25 | 0.46 | 0.10 | 1.00 | 0.47 | 0.25 | 0.42 |
| *pM* | 2.22 | 3.31 | 2.71 | 0.35 | 2.82 | 1.71 | 4.63 | 2.90 | 0.97 | 2.59 |
| *pMax* | 35.56 | 69.34 | 47.19 | 10.90 | 46.36 | 29.57 | 137.16 | 63.45 | 36.10 | 57.57 |
| *tM* | 16.71 | 18.65 | 17.40 | 0.60 | 17.25 | 17.97 | 19.85 | 18.99 | 0.60 | 19.04 |
| *tmax* | 22.74 | 25.69 | 23.68 | 1.01 | 22.99 | 23.71 | 27.40 | 25.58 | 1.09 | 25.51 |
| *tmin* | 10.93 | 12.48 | 11.62 | 0.56 | 11.53 | 11.86 | 13.54 | 12.68 | 0.60 | 12.65 |
| *tMax* | 32.11 | 39.49 | 35.45 | 2.18 | 35.32 | 33.68 | 38.57 | 35.67 | 1.79 | 35.38 |
| *tMin* | -3.18 | -0.91 | -2.39 | 0.75 | -2.88 | -4.44 | 0.72 | -1.43 | 1.84 | -1.01 |

*NOTE:* Min, minimum; Max, maximum; M, mean; SD, standard deviation; Md, median; N, sample size.


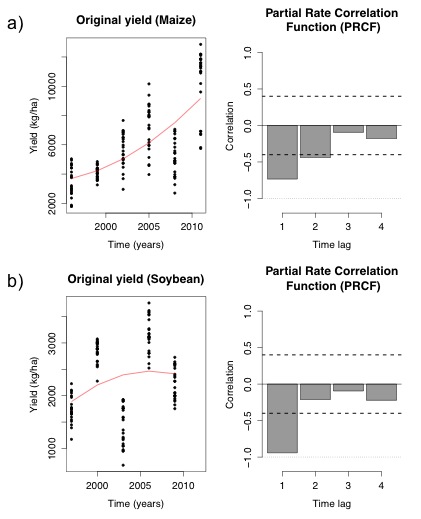


**Figure S1.** Diagnostic tools for the log-transformed time series of a) maize (*ZeaL*) and b) soyabean (*GlycL*) yield level. Its panels show the original time series and the Partial Rate Correlation Function (*PRCF*). Dotted lines indicate 95% confidence intervals ($2/\sqrt{n}$)


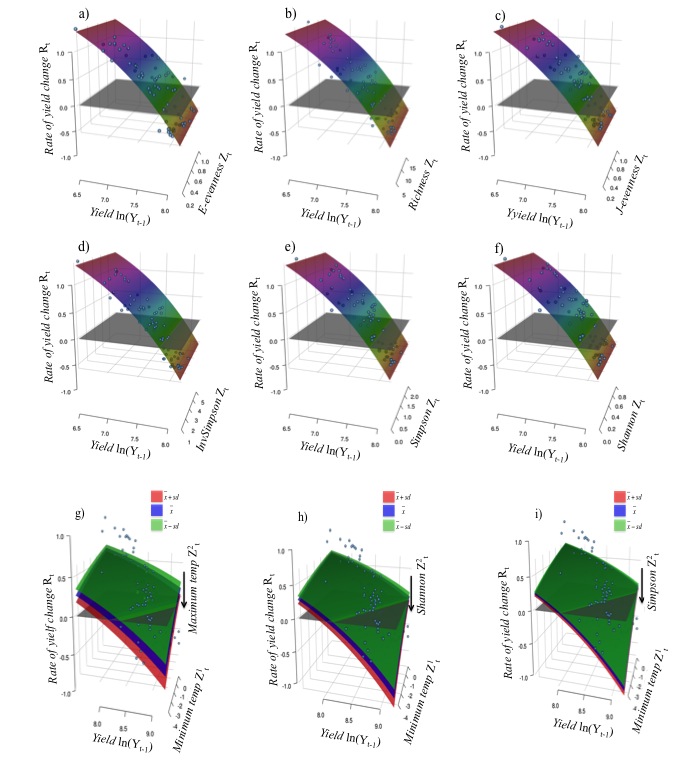


**Figure S2.** Annual rates of yield increase $R_{t}$ against the log-yield level $Y_{t-1}$ (with one year of delay) for: soyabean (*GlycL*; additive effects of weed diversity a-f) and maize (*ZeaL*; non-additive interaction effects of minimum temperature with: g) average maximum temperature (*tmax*/*tMin*), h) weed Shannon diversity index (*Shannon*/*tMin*) and i) weed Simpson diversity index (*Simpson*/*tMin*) interactions), where the external factor $Z_{t}$ perturbs the productivity function. Colours indicate the $R_{t}$ values. See Table S1 for description of models and Figure 2 for their graphs.

**Figure S3.** Pearson’s correlation coefficients of the weed community diversity indices for a) soyabean (*GlycL*) and b) maize (*ZeaL*).

**Figure S4.** Correlations between weed diversity indices (E-evenness, richness, Shannon, inverse Simpson) and the Hellinger-transformed weed species data (biomass) soyabean (*GlycL*). We used a threshold of for the correlations of interest. Large crabgrass (*Digitaria sanguinalis* L., DIGSA), red clover (*Trifolium pratense* L., ‘Michigan Mammoth Red’, TRIPR), garden yellowrocket (*Barbarea vulgaris* R. Br., BARVU), shepherd's purse (*Capsella bursa-pastoris* L., CAPBU), and giant foxtail biomass (*Setaria faberi* L., SETFA).

**Figure S5.** Correlations between weed diversity indices (Shannon and Simpson) and the Hellinger-transformed weed species data (biomass) for maize (*ZeaL*). We used a threshold of for the correlations of interest. Lambsquarters (*Chenopodium album* L., CHEAL), common dandelion (*Taraxacum officinale,* TAROF) and velvetleaf (*Abutilon theophrasti* Medik, ABUTH).

**Appendix A1.** We used the *diversity* function in the *BiodiversityR* package of R-Software to estimate diversity in each crop system with the estimated weed biomass. We calculated the species richness ($S$),Shannon–Wiener diversity index ($H=\sum_{i} p_{i}\ln(p_{i})$, where $p_{i}$ is the proportional biomass of species $i$ and $\ln$is the natural logarithm), both variants of Simpson's index (based on $D=\sum_{i} p_{i}^{2}$, Simpson index $1-D$ and Invsimpson index $1/D$), J-evenness ($H/\ln(S)$) and E-evenness ($\exp(H)/S$),

**Appendix A2.** External perturbations (by climate or weed diversity) could translate the conditional function curve ($R_{t}=f(Y_{t-d},Z)$) of maize and soybean yield with dynamic consequences. We modeled these effects by changes in each parameter of the equation (1). Changes in $r_{\max}$ (I) and $c$ (II) translate the curve moving it vertically and laterally, respectively, but do not influence its relative shape. However, perturbations in $a$ (III) influence the relative shape of the conditional production curve. Perturbations in the parameters and $a$ cause changes in the pattern of oscillation around equilibrium (its yield potential), but $c$ perturbations do not. The stability of the equilibrium point is determined by the slope of the function in the vicinity of equilibrium ($r_{\max}*a$): the steeper the slope, the less stable the equilibrium.
